# Circular RNA hsa_circ_0033144 (CircBCL11B) regulates oral squamous cell carcinoma progression via the miR-579/LASP1 axis

**DOI:** 10.1080/21655979.2021.1953214

**Published:** 2021-07-21

**Authors:** Wei Zeng, Mengmeng Guo, Lin Yao, Zhang Deng

**Affiliations:** a Department of Oral and Maxillofacial surgery, Meizhou People’s Hospital, Meizhou, Guangdong Province, China; b Stomatology Outpatient Department, Meizhou People’s Hospital, Meizhou, Guangdong Province, China

**Keywords:** CirBCL11B, miR-579, LASP1, OSCC

## Abstract

Oral squamous cell carcinoma is one of the most common malignant tumors of the head and neck. Increasing evidence suggests that various non-coding RNAs, such as circRNAs, are implicated in a myriad of biological processes supporting tumor progression. Recent studies have revealed that several circRNAs are dysregulated in oral squamous cell carcinoma (OSCC). However, their functional role in OSCC and the underlying mechanism remains to be further investigated. In this study, we aim to evaluate the biological role and survey the molecular mechanism of circBCL11B in regulating the progression of OSCC. We demonstrated that circBCL11B was significantly upregulated in OSCC tissues and cell lines, and the expression level was correlated with the malignancy. Silencing cirCBCL11B inhibited cell proliferation and migration, and also included cell apoptosis in OSCC cells. miR-145 was identified as a downstream target mediating the effect of circBCL11B by targeting LASP1. miR-145 negatively regulated LASP1 expression, which could be rescued by miR-145 inhibitor. Collectively, our study uncovered a functional role of circBCL11B/miR-579/LASP1 axis in OSCC, implying that targeting these molecules could be an intervention approach in OSCC treatment.

## Graphical Abtstract

**Figure:**
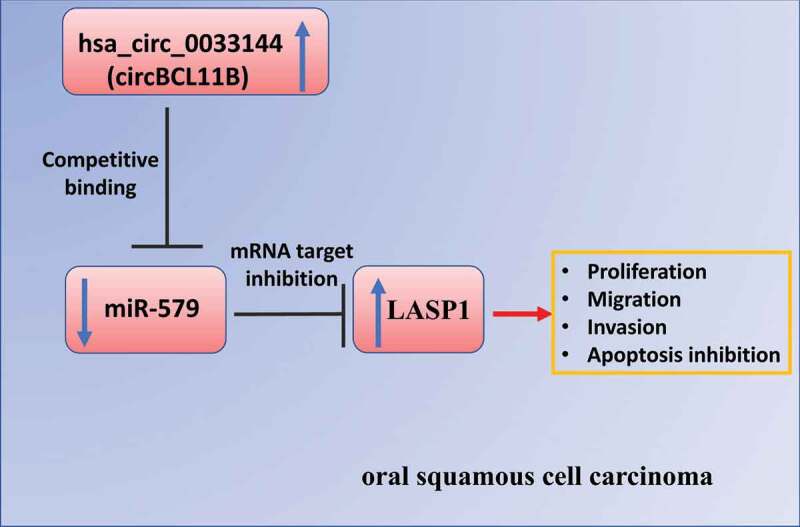

## Introduction

Oral cancer is a leading cause of morbidity and death in patients with head and neck tumors, and the treatment strategies are limited despite tremendous research efforts [1,2]. Surgical resection remains as the main treatment method at present, there are limited advances in its molecular diagnosis and the development of targeted therapies [3]. Understanding the mechanism underlying the occurrence and development of oral cancer is conducive to the development of biomarkers for early diagnosis and and targeted therapies.

CircRNAs (circular RNAs) are a new class of non-coding RNAs, which is usually produced by an anti-splicing event on one or both exons, resulting in covalently closed circular RNA molecules [1,4]. Due to the closed-loop structure, circRNAs are not easily digested by exonucleases and are more stable than linear non-coding RNAs [5]. Previous studies have shown that a growing body of circRNAs are implicated in a wide spectrum of biological processes and the pathological conditions. In many cases, circRNAs act as a miRNA sponge, regulating the splicing event or the activity of miRNAs on the downstream targets [6]. It has been pointed out that hsa_circ_0033144(circBCL11B) is upregulated in oral squamous cell carcinoma [7].

MicroRNAs are short non-coding RNA which often target mRNAs to regulate their transcription or stability [8,9]. Accumulating evidence suggests that microRNAs functions as a downstream effector of lncRNAs or circRNAs in different pathophysiological conditions including cancers [10,11]. Among them, miR-579 is identified as a novel tumor suppressor and a novel target in spongioblastoma by regulating the PI3K/AKT pathway [12].

It has also been reported that miR-579 regulates the development of osteosarcoma by modulating MMP13 expression [13]. In addition, miR-579 was reported to form a complex with miR-221, miR-125b and specific RNA-binding proteins (RBPs) to target 3ʹUTR of TNFα mRNA, which impairs the protein synthesis by interfering with the translation [14]. Aberrant expression of miR-579 has been also reported in a variety of tumors and diseases [15–17], however, its potential involvement in oral squamous cell carcinoma (OSCC) remains to be studied. In this study, we evaluated the biological role and survey the molecular mechanism of circBCL11B in OSCC. Our data showed a significant upregulation of circBCL11B in OSCC tissues and cell lines. Silencing cirCBCL11B inhibited the malignant phenotype of OSCC cells, and induced cell apoptosis. miR-145 was identified as a downstream target mediating the effect of circBCL11B by targeting LASP1. In summary, our study unveiled a functional role of circBCL11B/miR-579/LASP1 axis in OSCC, implying that targeting these molecules could be novel approach in OSCC treatment.

## Materials and methods

### Cell culture

OSCC cell lines including Cal-27, FADU, OECM1, SAS, HSC3, and SCC9 were obtained from American Type Culture Collection. Normal oral keratinocytes (NHOK) were acquired from Shanghai Henlius Biotech. All the cells were maintained in DMEM (Gibco,USA) medium containing 10% FBS (Gibco,USA) and 1% penicillin/streptomycin (Hyclone, USA) in a humidified incubator under the condition of 37°C and 5% CO2. The cells were subcultured every 2 or 3 days in fresh medium. Cells were harvested for subsequent experiments in exponential growth state.

### Clinical sample collection

A total number of 50 patients diagnosed with OSCC were recruited from the Meizhou People’s Hospital, Guangdong Province, China. Patients who participated in this study had not received chemotherapy or radiation before surgery. All samples were collected from primary tumor tissues of OSCC patients and the adjacent normal oral mucosal tissue near the tumor resection margin by surgery. The collected tissues were snap-frozen in liquid nitrogen for subsequent use. This study was approved by the ethic committee of Meizhou People’s Hospital (2019–101,206). All patients were informed of the details of the experiment and signed the informed consent.

### Cell transfection

SiRNA negative control (si-NC), si-circBCL11B RNAs, miR-579 mimic, and miR-579 inhibitor was transfected into Cal-27 and SCC9-7 cells by Lipofectamine 3000 (Thermo Fisher Scientific, USA) following the manufacturer’s protocol. Briefly, cells were seeded in 6-well plates at a density of 5×10^5 cells/well. Twenty-four hours later, 100 nm of each molecule was added into 100 µl Opti-MEM® I Reduced-Serum Medium (Invitrogen, Carlsbad, CA), and then 6 µL Lipofectamine 3000 reagent was added for 10 min incubation at room temperature. The mixture was added dropwise to the cell culture. Fresh medium was replaced 6 h after transfection and cells were harvested for subsequent experiments after 48 h. siRNA sequences are as follows:

si-circBCL11B#1: 5ʹ-ATTGCAGCAGAGGCTGACCAT-3ʹ;

si-circBCL11B#2: 5ʹ-TGCAGCAGAGGCTGACCATGT-3ʹ;

si-circBCL11B#3:5ʹ-CATTGCAGCAGAGGCTGACCA-3ʹ;

siNC: 5ʹ-TTCTCCGAACGTGTCACGT-3ʹ

miR-579 mimic, and miR-579 inhibitor was purchased from GenePharma (Shanghai, China).

### CCK-8 cell proliferation assay

OSCC cells were seeded into 96-well plates at a density of 2 × 10^3^ cells/well, respectively. Lipofectamine^TM^3000 (Thermo Fisher Scientific, USA) was utilized to perform transfection as described above. After transfection, cells were further cultured for 24, 48, 72, or 96 hours. Ten microliter CCK8 reaction solution (Solarbio, CA1210, Beijing, China) was added to the cell culture at indicated time point and incubated for 1 h in a humidified cell culture incubator. The light absorption value (OD value) in each condition was captured at 450 nm wavelength on a microplate reader (Thermo Fisher Scientific).

## Real-time quantitative PCR

Total RNAs in tissues or cells were purified by Trizol reagent (15,596,026, Thermo Fisher Scientific, USA). 1 µg total RNA was reverse transcribed into cDNA by Prime ScriptTMRT Master Mix (Takara, USA). The resulted cDNA was diluted to 40 ng/μL and analyzed in the FTC-3000p Q PCR system (Funglyn Biotech, Canada) using SYBR premix EX TAQ II kit (RR820A, Takara, Dalian, China). The PCR cycling condition used: 95℃2 min, 40 cycles of 95℃ 30 s, 60℃ 30 s and 72℃ 60 s, with signal detection at the end of each cycle. Finally, the 2–∆∆Ct method was used to analyze the relative expression level and GAPDH was used as the internal reference gene. The sequences of primers used in this assay were listed at below:

circBCL11B: (Forward:5ʹ-CATTGCAGCAGAGGCTGACCA-3ʹ; reverse: 5′-ACT GAAATGCTAATGTGTGGC −3′);

BCL11B (forward: 5′-ATGTCCCGCCGCAAACAGG-3′; reverse: 5′-GGCTCGGAC ACTTTCCTGAGC-3′);

miR-579 (forward: 5′- GTGCAGGGTCCGAGGT −3′; reverse: 5′- TTAACAAAGTG CTCATAGTGC −3′);

GAPDH (forward: 5′- AAGGTCGGAGTCAACGGATT-3′;reverse:5′CTGGAAGA TGGTGATGGGATT T-3′).

## Western blotting analysis

OSCC cells were harvested at 48 h after transfection and then lysis buffer with protease inhibitor (MedChemExpress, USA) was employed to extracting the total protein. Protein concentration was quantified by a BCA Protein assay kit (Solarbio, Beijing, China). Twenty ug total protein was loaded for SDS-PAGE and separated protein in SDS-PAGE gel was transferred onto a PVDF membrane (BioRed, USA). After blocking with 5% skimmed milk for 1 h, the membrane was then incubated with primary antibodies overnight at 4°C. The membrane was washed 3 times with TBST buffer and further incubated with HRP-linked secondary antibody. The protein bands were visualized using an enhanced chemiluminescence kit (Santa Cruz, TX, USA) and photographed on a gel imager system (Bio-Rad). Finally, protein levels were analyzed using the Image-Pro Plus Image analysis system.

### Clonogenic assay

Cells were seeded in 6-well plates for 10 days after transfection for 48 h. The culture medium was changed every 3 days during the period. After 10 days, cells were fixed with 4% paraformaldehyde at room temperature for 10 mins and stained with Giemsa reagent (Giemsa Stain Kit, Abcam ab150670) for 20 mins. Subsequently, the number of colonies was counted and the morphology of the colonies was photographed under Leica AM6000 microscope.

### Transwell migration and invasion assays

Cell invasion ability was monitored by transwell assay with matrigel. Transwell chambers (Sigma, Germany) are pre-coated with matrigel (Sigma, Germany). Cells with different treatments were trypsinized and resuspended in serum-free medium. 1 × 10^5^ cells were inoculated into the transwell upper chamber in serum-free medium and 500 μL of 10% serum-containing medium was added to the lower chamber. After 16 hours, culture medium was discarded and the cells were fixed with 4% paraformaldehyde at room temperature for 10 mins and stained with 0.5% crystal violet (Sigma, Germany) for 20 mins. Cells were photographed under Leica AM6000 microscope and the number of invading cells was counted. Cell invasion ability was measured by the number of cells penetrating the membrane. Cell migration ability was examined by transwell assay without matrigel. The rest of the procedures were similar to the cell invasion assay described above.

### Dual luciferase reporter assay

Wild-type circBCL11B sequence, mutant circBCL11B sequence, wild-type LASP1 3ʹUTR and mutated LASP1 3ʹUTR were cloned into PmirGLO reporter vector expressing firefly luciferase respectively (Promega, E1330). The reporter plasmid and Renilla luciferase (hRlucneo) control plasmid were co-transfected into cells with either miRNA mimic or inhibitor in a 12-well plate (1 × 10^5 cells/well) using Lipofectamine 3000 reagent. Forty-eight hours after transfection, the relative luciferase activities were measured using Dual-Luciferase Reporter Assay Kit (Promega, E1910) on a luminescence microplate reader (Infinite 200 PRO; Tecan). The relative firefly luciferase activity in the reporter plasmid was normalized to that of Renilla luciferase (hRlucneo) control plasmid.

### Flow cytometer assay

OSCC cells under different treatments were trypsinized and washed twice with 1xPBS, and resuspended in the staining buffer. The detection of cell apoptosis was performed using the apoptosis kit (BD Biosciences, PharMingen, San Jose, CA, USA) according to the manufacturer’s instructions. In brief, 5 μL Annexin V-FITC and 5 μL PI were added to the 500 μL cell resuspension conatining 0.5 million cells and incubated for 30 mins in the dark. Stained cells were centrifuged and washed twice with 1xPBS and resuspended in 400 μL 1xPBS. The percentage of apoptotic cells was detected by BD FACS CantoTM II Flow Cytometer (BD Biosciences).

### EDU incorporation assay

Cells were seeded 96-well plates and transfected with siRNA for 48 h. Click-iT™ EdU Cell Proliferation Kit for Imaging, Alexa Fluor™ 555 dyeEDU solution (C10338, Thermo Fisher Scientific, USA) was used to detect cell proliferation. Prewarmed the 2X EdU solution was added in an equal volume of the cell culture medium, and incubated for 2 h. Cells were fixed with 50 µL of 3.7% formaldehyde in PBS for 15 min at room temperature. After the removal of the fixative and the cells were washed twice with 100 µL of 3% BSA in PBS. Then, 50 µL of 0.5% Triton® X-100 in PBS was added to each well for 20 min incubation. 1 x Click-iT® reaction cocktail was prepared and added to the cells for 30 mins. The staining cocktail was removed and the cells were further washed twice with 100 µL of 3% BSA in PBS. 500 nM DAPI in PBS was used for nuclear staining and the cell images were captured under Leica AM6000 microscope.

### Nuclear and cytoplasmic fractioning

For nucleoplasm fraction experiment, the nuclear and cytoplasmic fraction was extracted using NE-PER™ Nuclear and Cytoplasmic Extraction Reagents (Thermo Fisher Scientific, 78,833), and the total RNA in each fraction was purified using Trizol reagent (Invitrogen, 15,596,026) according to the manufacturer’s protocol. An equal amount of cells were used for total cell lysate RNA extraction, which serves as the total cellular RNA level control for normalization. The extracted RNA was quantified by RT-qPCR.

### RNA pull-down assay

Cells lysates were collected by IP lysis buffer (Beyotime, P0013) and were incubated biotinylated-miR-579 oligo and Control oligos. Ten percent of the lysates was saved as the input. The mixture was further incubated with M-280 streptavidin magnetic beads (Sigma-Aldrich, 11205D) at 4°C shaking overnight. A magnetic bar was used to pull down the magnetic beads and associated nucleic acids, then the samples were washed 4 times with high salt wash buffer. Both the input and the elutes from the pull-down were purified with Trizol reagent (Invitrogen, 15,596,026) according to the manufacturer’s protocol. The reverse transcription was carried out using Superscript III transcriptase (Invitrogen, 18,080,093) and quantitative RT-PCR analysis was performed using Maxima SYBR Green/ROX qPCR Master Mix (Thermo Fisher Scientific, K0221) on a LightCycler® 96 real-time PCR system (Roche).

## Statistical analysis

SPSS 19.0 software was used for statistical analysis and each experiment was performed 3 times independently. The statistical difference between two groups was compared using unpaired student’s t tests. Comparisons among multiple groups were analyzed using one-way analysis of variance (ANOVA) with Tukey’s post hoc test for pairwise comparison. Comparisons of data at multiple time points were examined using two-way ANOVA. Kaplan Meier Curve and log-rank test were used to compare the cumulative survival rates. Data were reported as mean ± SEM. P < 0.05 was considered as statistically significant.

## Results

It has been pointed out that hsa_circ_0033144 (circBCL11B) is upregulated in oral squamous cell carcinoma (OSCC) [7]. However, whether circBCL11B regulates the progression of OSCC and its the molecular target remain to be determined. In this study, we investigated the biological role and studied the molecular mechanism of circBCL11B in OSCC. Our data showed a significant upregulation of circBCL11B in OSCC tissues and cell lines. Silencing cirCBCL11B inhibited the malignant phenotype of OSCC cells, and induced cell apoptosis. miR-145 was identified as a downstream target mediating the effect of circBCL11B by targeting LASP1. In summary, our study unveiled a functional role of circBCL11B/miR-579/LASP1 axis in OSCC, implying that targeting these molecules could be novel approach in OSCC treatment.

## CircBCL11B is substantially upregulated in oral squamous cell carcinoma tissues and cell lines

**Figure 1.**
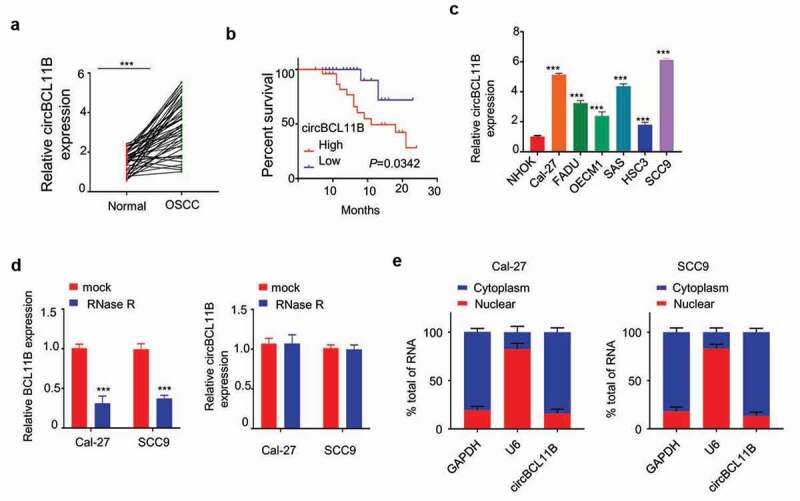
circBCL11B expression is remarkably increased in oral squamous cell carcinoma tissues and cell lines

The expression of circBCL11B in 50 paired OSCC tissues and adjacent normal oral mucosal tissues was quantified by qRT-PCR. As the result showed, circBCL11B expression level was remarkably higher in OSCC tissues (Figure 1(a)). And Kaplan Meier Curve showed that high circBCL11B expression was associated with shorter overall survival (Figure 1(b)). Furthermore, circBCL11B expression was upregulated in various OSCC cell lines (Cal-27, FADU, OECM1, SAS, HSC3, SCC9) as compared to normal oral keratinocytes (NHOK) (Figure 1(c)). We next validated that circBCL11B has a circular structure as circBCL11B was resistant RNase R digestion while BCL11B was degraded (Figure 1(d)). Next, we performed nuclear/cytoplasmic separation assay in CAL-27 and SCC9 cells to identify the cellular sublocalization of circBCL11B. The results of qRT-PCR showed that cirCBCL11b was mainly localized in the cytoplasm (Figure 1(e)).

**A** circBCL11B expression in 50 couples of oral squamous cell carcinoma tissues and cell lines were detected by qRT-PCR assay (p < 0.001).**B** Kaplan-Meier survival curve were used to evaluate overall survival time in OSCC patients with low cirCBCL11b expression (n = 25) and high cirCBCL11b expression (n = 25) (p < 0.001). **C** The mRNA levels of circBCL11B in a series of OSCC cell lines (Cal-27, FADU, OECM1, SAS, HSC3, SCC9) and normal oral keratinocytes (NHOK) were showed by qRT-PCR (p < 0.01). **D** The expression levels of BCL11 B and cirCBCL11 B in CAL-27 and SCC9 cells after RNase R treatment were measured by qRT-PCR. **E** Nucleoplasmic separation assay showed the cell sublocalization of circBCL11B, which was quantified by qRT-PCR (p < 0.01). Data are summary of three independent experiment.

To further study the relationship between circBCL11B expression and clinical features, the median expression value of cirCBCL11B expression level in OSCC tissues was set as cutoff value, and 50 OSCC patients were divided into two groups: the high expression group (n = 25) and the low expression group (n = 25). The chi-square test was used to determine the relationship between the expression level of cirCBCL11B and the pathological data of OSCC. The results showed that high cirCBCL11B expression was correlated with tumor size, TNM stage, and distal metastasis, and the difference was statistically significant (P < 0.05), but it was not related to the patient’s age, gender, or tumor differentiation (Table 1).

**Table 1.** Association between circCBCL11b expression and clinical pathologic parameters in OSCC patients

Chinicopathological characteristics		expression		total	χ^2^	*P*
		low	high			
Age (year)					0.321	0.571
	≤60	13	11	24		
	>60	12	14	26		
Tumor differentitation					1.389	0.239
	Low	7	11	18		
	High	18	14	32		
Gender					0.321	0.571
	Female	14	12	26		
	male	11	13	24		
TNM stage					3.945	0.047
	Ι/II	15	8	23		
	III/IV	10	17	27		
Tumor size					5.195	0.023
	<3 cm	18	10	28		
	>3 cm	7	15	22		
Distal metastasis					4.16	0.041
	M0	19	12	31		
	M1	6	13	19		

## Silencing cirCBCL11B suppressed the malignant activity of OSCC and induced cell apoptosis

**Figure 2.**
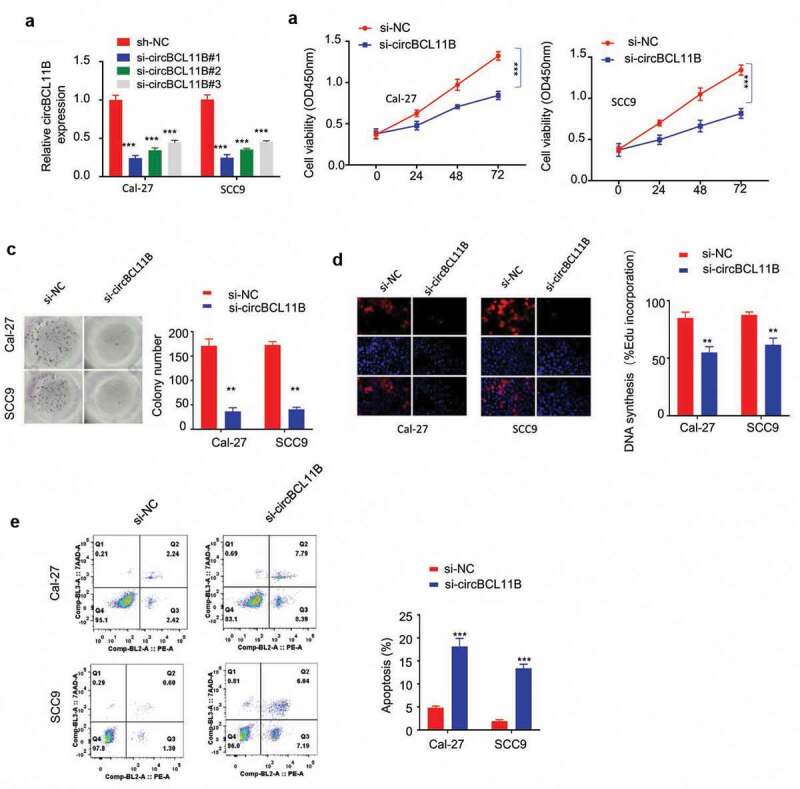
circBCL11B silencing restricts cell progression and induced cell apoptosis A

To further explore the functional role of cirCBCL11B in OSCC progression, we constructed three siRNA oligos to silence cirCBCL11B and the knockdown efficiency was confirmed by qRT-PCR in Cal-27 and SCC9 cells (Figure 2(a)). The transfection of cirCBCL11B in OSCC cell lines by si-cirCBCL11B#1 showed the best knockdown efficiency. Functionally, cirCBCL11B silencing suppressed OSCC cell viability as detected by CCK-8 cell proliferation assay (Figure 2(b)), clonogenic assay (Figure 2(c)) and EDU incorporation assay (Figure 2(d)). We then examined whether circBCL11B affected apoptosis in OSCC cells. Flow cytometry results showed that cell apoptosis was significantly induced by circBCL11B silencing (Figure 2(f)). Therefore, cirCBCL11B is indispensable for the survival and proliferation of OSCC cells.

Knockdown effect of siRNA oligos targeting circBCL11B in Cal-27 and SCC cells (p < 0.01). **B-D** Cell were transfected with siRNA targeting circBCL11B. CCK-8 cell proliferation assay (B), clonogenic assay (C), and EDU incorporation assay (D) demonstrated that knockdown circBCL11B inhibited cell proliferation in OSCC cells (p < 0.01). **E** The apoptosis level of different groups of CAL-27 and SCC9 cells (si-NC, si-circCBCL11b) was detected by flow cytometry (p < 0.01). Data are summary of three independent experiment.

## Silencing circBCL11B inhibits migration and invasion of oral cancer cells

**Figure 3.**
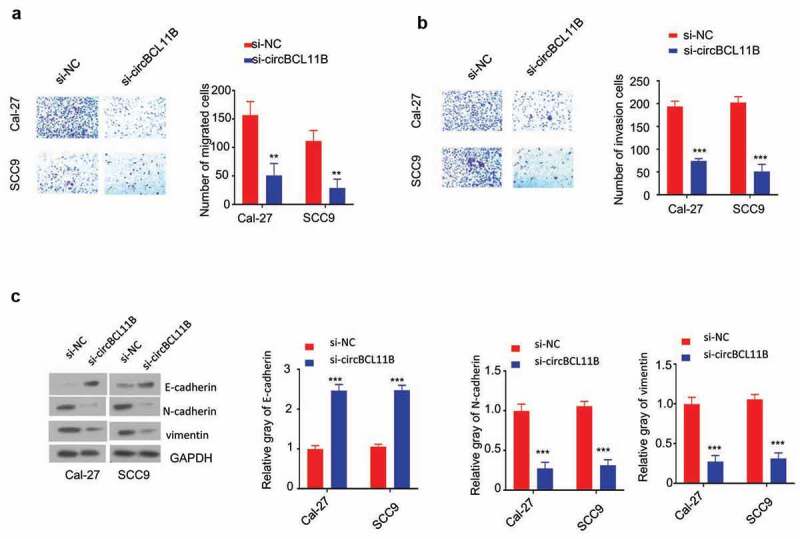
Knockdown of circBCL11B inhibited cell migration and invasion in vitro. A-B Transwell assays detected cell migration (a) and invasion (b) in Cal-27 and SCC9 cells (p < 0.01). **C**. The protein levels of E-cadherin, N-cadherin, vimentin in CAL-27 and SCC9 cells were quantified by western blotting after transfecting with si-NC, si-circCBCL11b. Data are summary of three independent experiment

Transwell assay without matrigel was performed in Cal-27 and SCC9 cell to determine cell migration after silencing circBCL11B (Figure 3(a)). Cell invasion assay was performed in tranwell coated with Matrigel (Figure 3(b)). The knowckdown of circBCL11B significantly inhibited the cell migration and invasion. As cell migration and invasion are closely connected with epithelial-mesenchymal transformation (EMT), we next examined the changes of EMT markers. EMT leads to loss of epithelial phenotypes and the decreased level of E-cadherin would lead to the impaired cell adhesion, which enables cells to acquire migratory characteristics. Meanwhile, cells gain mesenchymal phenotypes, such as the increased expression of Vimentin and N-cadherin. Western blot analysis revealed that circBCL11B knockdown up-regulated the expression of E-cadherin protein, whereas N-cadherin, vimentin protein expression is down-regulated (Figure 3(c)). Taken together, circBCL11B promotes cell migration and invasion by regulating EMT in OSCC cells.

## CircBCL11B interacts with miR-579 and regulates its expression

**Figure 4:**
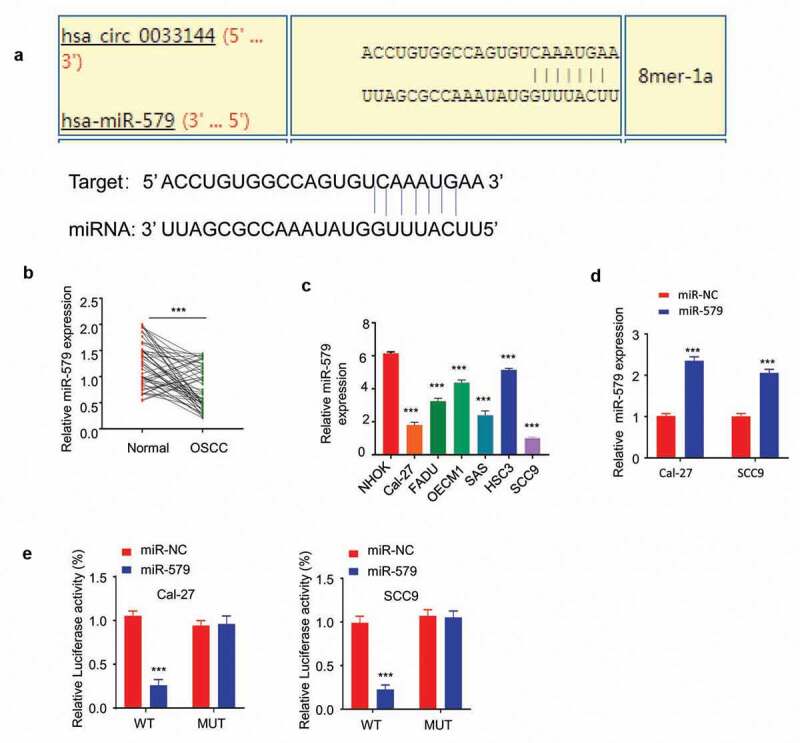
circBCL11B interacts with miR-579 and regulate its expression

We next investigated the potential downstream target of CircBCL11B. miR-579 may act as a target of circBCL11B as miR-579 was predicted to interact with circBCL11B by Circinteractome analysis (Figure 4(a)). Also, the expression level of miR-579 was closely related to oral cancer progression. qRT-PCR results showed that the expression of miR-579 was drastically reduced in cancer tissues (Figure 4(b)). Similarly, down-regulation of miR-579 was identified in OSCC cells (Cal-27, SCC9) as compared with normal oral keratinocytes (NHOK) (Figure 4(c)). miR-579 mimic was constructed and its transfection could increase miR-579 level as detected by qRT-PCR (Figure 4(d)). We then performed dual luciferase reporter assay to verify the functional interaction between miR-579 and circBCL11B. Overexpression of miR-579 inhibited luciferase activity of wild-type cirCBCL11B reporter in CAL-27 and SCC9 cells, but not in the mutated reporter (Figure 4(e)). Thus, we considered that miR-579 may as the potential target of circBCL11B.

**A** Circinteractome analysis showed that circBCL11B contained a binding site of miR-579. **B** miR-579 in OSCC cells showed downregulation as compared with normal cells (p < 0.01). **C** The transfection effect of miR-579 mimic was demonstrated by qRT-PCR (p < 0.01). **D** Dual-luciferase reporter assay measured the luciferase activity of wild-type circBCL11B vector and mutation circBCL11B vector in Cal-27 and SCC9 cells after transfecting with miR-579 mimic. Data are summary of three independent experiment. **E** miR-579 expression in 50 pairs of oral cancer and normal tissues was detected by qRT-PCR (p < 0.001).

## CircBCL11B regulate LASP1 via miR-579

**Figure 5.**
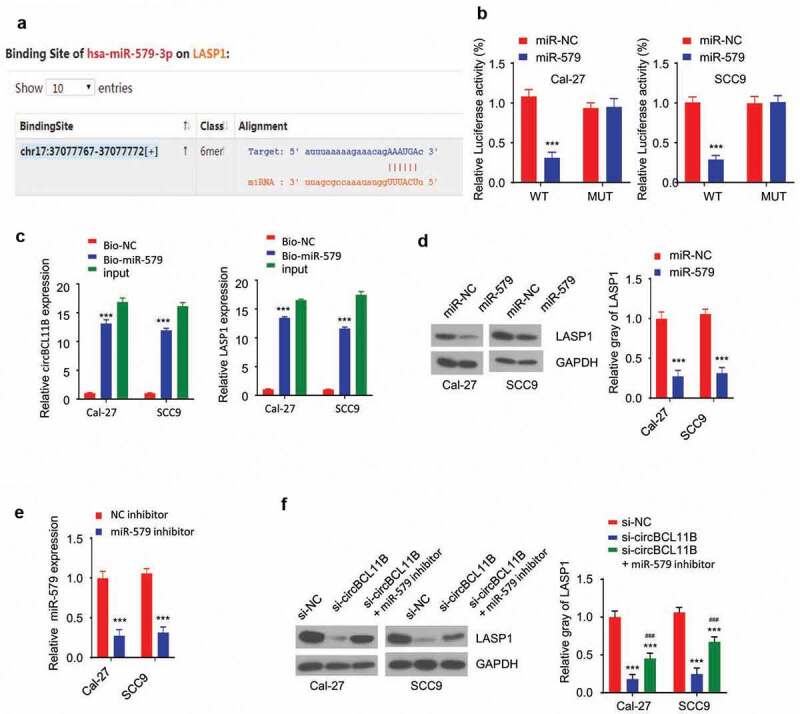
circBCL11B regulates LASP1 expression via miR-579

We next aimed to find the potential target gene of miR-579. Starbase predicted the presence of the miR-579 binding site in the 3 ‘UTR of LASP1, so LASP1 may be a potential target gene of miR-579 (Figure 5(a)). The dual luciferase reporter assay showed that miR-579 overexpression inibited LASP1 3ʹUTR-WT sequence reporter, but no inhibition was observed in LASP1 3ʹUTR-MUT sequence (Figure 5(b)). Moreover, RNA pull-down assay showed that biotinylated-miR-579 oligo interacted with both cirCBCL11b and LASP1 as it significantly enriched both molecules in Cal-27 and SCC9 cells (Figure 5(c)). miR-579 overexpression reduced the protein level of LASP1 in OSCC cells (Figure 5(d)). To further confirm the role of miR-579 in LAPS regulation, we used a miR-579 inhibitor which markedly downregulated miR-579 expression after transfection (Figure 5(e)). Western blotting results showed that cirCBCL11b silencing reduced the protein expression level of LASP1, which was rescued by co-transfected with miR-579 inhibitor. Together, these results indicate that miR-579 functions as a downstream effector of CircBCL11B to regulate LASP1.

**A** Starbase predicted that miR-579 may bind to the 3 ‘noncoding region of LASP1. **B** Dual-luciferase reporter assay showed the luciferase activity of LASP1 3ʹUTR-WT and LASP1 3ʹUTR-MUT vector after transfecting with miR-579 mimic. **C** RNA pull-down was performed to detect the binding between miR-579 and circBCL11B or LASP1 (p < 0.01). **D** Western blotting showed the protein level of LASP1 in miR-579 overexpressed cells. **E** The effect of miR-579 inhibitor was detected by qRT-PCR (p < 0.01). **F** Protein levels of LASP1 in OSCO cells (si-NC, si-circBCL11B, si-circBCL11B+miR-579 inhibitor) were detected by Western blotting. Data are summary of three independent experiment.

## Discussion

circRNAs, as a novel class of endogenous RNA, is lowly expressed in eukaryotic transcriptome [18]. In contrast to lncRNAs and miRNAs, circRNAs have a covalently closed structure, instead of 5ʹ-3ʹ polarity and a polyadenylated tail [19]. Accumulating studies have shown that various circRNAs are implicated in physiological conditions and diseases. In particular, some circRNAs act as tumor suppressors or tumor promoting factors [20,21]. Our study demonstrated that cirBCL118 may serve as a potential biomarker or therapeutic target for OSCC: (1) circBCL11B is upregulated in both OSCC tissues and cell lines; (2) cirBCL11B expression level informs the porgonsis in OSCC patients; (3) circBCL11B silencing arrests OSCC cell proliferation, invasion, and migration; (4) Silencing circBCL11B promotes cell apoptosis.

Several circRNAs have been implicated in OSCC progression by modeling different pathways. For example, circ_0000467 was reported to regulate colorectal cancer development via miR-382-5p/EN2 axis [22]. In non-small cell lung cancer cells, circular RNA circ_0001287 suppresses the proliferation, metastasis, and radiosensitivity by sponging microRNA miR-21 and up-regulating phosphatase and tensin homolog expression [23]. Furthermore, circular RNA ZNF609 functions as a ceRNA in regulating E2F transcription factor 6 by targeting microRNA-197-3p to promote cervical cancer progression [24]. Our studies add further evidence that circBCL11B function as a tumor-promoting factor by targeting miR-579/LASP1 axis in OSCC cells. Together, these studies and our results further highlighted the diversified functional roles of circRNAs in different types of cancers.

miRNAs are a class of small single-stranded non-coding RNAs, regulating the transcription of mRNAs or its stability after transcription [25]. Diverse miRNAs are involved in a range of physiological activities, including metabolism, cell differentiation, and tumorigenesis [26–28]. Our study further delineated the molecular mechanism of circBCL11B in regulating OSCC by targeting miR-579/LASP1 axis. miR-579 is a downstream effector of circBCL11B, which negatively impacts on the expression of LASP1. Multiple reports have shown that LASP1 promotes invasion and metastasis in a variety of tumors including pancreatic cancer, colorectal cancer, and medulloblastoms [29–31]. Consistently, our study showed that silencing circBCL11B leads to the upregulation of miR-579 and the downregulation of LASP1, which may account for the impaired survival and enhanced apoptosis phenotype. Therefore, previous studies and our results both indicates that LASP1 acts as a tumor-promoting gene.

Importantly, we showed that by RNA pull-down assay miR-579 can physically interact with both circBCL11B and LASP1 in OSCC cells. This support a ceRNA regulatory model of miR-579 mediating the effect of circBCL11B. Similarly, miR-579-3p has been reported to regulate the development of squamous cell lung carcinoma by targeting macrophage scavenger receptor 1 (MSR1) [17]. miR-579-3p also controls melanoma progression and its sensitivity to target therapy by targeting the 3′UTR of two oncoproteins: BRAF and an E3 ubiquitin protein ligase, MDM2 [32]. Our study provided novel evidence of miR-579 in regulating OSCC progression, suggesting the miR-579 family could be potential therapeutic targets.

## Conlusions

Taken together, our study provided evidence that circBCL11B interacts with miR-579 to regulate the expression level of LASP1. miR-579 is downregulated in OSCC tissues and cell lines, which may result from the upregulation of circBCL11B. miR-579 overexpression and circBCL11B silencing could reduce protein level of LASP1 in Cal-27 and SCC9 cells. These results suggest that circBCL11B antagonize the activity of miR-579 in modulating LASP1. Future work will be needed to further investigate the roles of circBCL11B/miR-579/LASP1 axis in tumorigenesis using animal model.
